# Characterizing Spatiotemporal Dynamics of CH_4_ Fluxes from Rice Paddies of Cold Region in Heilongjiang Province under Climate Change

**DOI:** 10.3390/ijerph16050692

**Published:** 2019-02-26

**Authors:** Tangzhe Nie, Zhongxue Zhang, Zhijuan Qi, Peng Chen, Zhongyi Sun, Xingchao Liu

**Affiliations:** 1 School of Water Conservancy and Civil Engineering, Northeast Agricultural University, Harbin 150030, China; nietangzhe@neau.edu.cn (T.N.); liuwanning@neau.edu.cn (P.C.); neauliuxing@163.com (X.L.); 2 Key Laboratory of Agricultural Water Resource Use, Ministry of Agriculture, Harbin 150030, China; 3 Graduate School of Agriculture, Hokkaido University, Sapporo 060-8589, Japan; sunzy025@env.agr.hokudai.ac.jp

**Keywords:** climate change, rice paddies of cold region, CH_4_ fluxes, spatiotemporal distribution, DNDC model

## Abstract

Paddy fields have become a major global anthropogenic CH_4_ emission source, and climate change affects CH_4_ emissions from paddy ecosystems by changing crop growth and the soil environment. It has been recognized that Heilongjiang Province has become an important source of CH_4_ emission due to its dramatically increased rice planting area, while less attention has been paid to characterize the effects of climate change on the spatiotemporal dynamics of CH_4_ fluxes. In this study, we used the calibrated and validated Long Ashton Research Station Weather Generator (LARS-WG) model and DeNitrification-DeComposition (DNDC) model to simulate historical and future CH_4_ fluxes under RCP 4.5 and RCP 8.5 of four global climate models (GCMs) in Heilongjiang Province. During 1960–2015, the average CH_4_ fluxes and climatic tendencies were 145.56 kg C/ha and 11.88 kg C/ha/(10a), respectively. Spatially, the CH_4_ fluxes showed a decreasing trend from west to east, and the climatic tendencies in the northern and western parts were higher. During 2021–2080, the annual average CH_4_ fluxes under RCP 4.5 and RCP 8.5 were predicted to be 213.46 kg C/ha and 252.19 kg C/ha, respectively, and their spatial distributions were similar to the historical distribution. The average climatic tendencies were 13.40 kg C/ha/(10a) and 29.86 kg C/ha/(10a), respectively, which decreased from west to east. The simulation scenario analysis showed that atmospheric CO_2_ concentration and temperature affected CH_4_ fluxes by changing soil organic carbon (SOC) content and plant biomass. This study indicated that a paddy ecosystem in a cold region is an important part of China’s greenhouse gas emission inventory in future scenarios.

## 1. Introduction

Paddy fields are important source of greenhouse gas emissions, and CH_4_ emissions from paddy fields accounts for about 20% of global anthropogenic CH_4_ emissions annually [1]. According to the Food and Agricultural Organization (FAO), due to the increasing human population, the rice yield needs to increase by 40% by the end of the 2030s [2]. This suggests that paddy fields will continue to be an important anthropogenic CH_4_ source worldwide [3].

The CH_4_ emissions from the paddy fields mainly depends on a series of biochemical processes in the soil such as CH_4_ production, CH_4_ oxidation, and pathways of gas transportations such as plant-mediated transport, molecular diffusion, and ebullition [4,5]. These processes are affected by many environment factors, and climatic factors are among the key controlling and driving factors [6]. Climate change affects CH_4_ emissions from paddy ecosystems by changing crop growth and the soil environment. Changes in temperature change the activity of methanogens in soil [7]. Different levels of rainfall change the soil moisture and aeration status, and further affect CH_4_ production and oxidation [8]. Increasing atmospheric CO_2_ concentration contributes to crop biomass accumulation and provides more carbon sources for CH_4_ production in soil [9,10]. Changes in solar radiation will affect crop growth through crop photosynthesis, thereby affecting the crop transmission of CH_4_ [11,12]. Increased atmospheric CO_2_ concentration due to human activities will have a greater impact on climate change in the 21^st^ century [13,14]. According to the Intergovernmental Panel on Climate Change (IPCC) Fifth Assessment Report, significant warming was estimated under different representative concentration paths (RCPs) during the 21st century by 1.3~6.1 °C [15]. The temperature in China was expected to increase at a rate of 0.06~0.63 °C per decade, and rainfall was also expected to increase by 0.52~1.85 per decade [16]. These changes of climatic factors would definitely affect the CH_4_ emissions from paddy fields.

Several studies on the effects of climate factors on the spatiotemporal distributions of CH_4_ emissions from paddy fields can be found in the literature, which mostly have been concentrated in Asian countries. For example, the study by Katayanagi et al. [8] indicated a decreasing trend of CH_4_ emission from rice paddies from north to south in Japan, considering organic carbon input, water management regimes, and the drainage rates of Japan’s seven administrative regions from 1990 to 2012. Minamikawa et al. [17] pointed out that the CH_4_ emissions from the rice paddies of six experiment sites in central Thailand increased by different ranges, and also showed a big spatial distribution from 2001 to 2060 under four RCPs under different water management practices. In China, Li et al. [18] found that Chinese fields emitted 6.4~12.0 Tg C per year within a continuous flooding scenario under 1990 climate land-use conditions by modeling CH_4_ emissions using DNDC, while southern China showed larger CH_4_ emissions than other parts, and in another paper, an increasing trend of CH_4_ emission of rice paddies in China from 2000 to 2020 by different management scenarios was also found [19]. However, no big changes of CH_4_ emissions from rice fields in China between 1991–2010 was found in a study by Verburg et al. [20]. Meanwhile, Zhang et al. [21] modeled CH_4_ emissions from irrigation rice cultivation of five regions in China and predicated that the CH_4_ emission amount from 2010 to 2050 would increase by 1.2 kg ha^−1^ yr^−1^, and that the increase in northeastern China was more significant than in other areas. At a smaller scale, Zhang et al. [22] characterized the spatial dynamics of CH_4_ emissions from rice paddies of Sanjiang Plain in the years 1990, 2000, and 2010; he found that big spatial differences and annual average differences of CH_4_ fluxes existed, due to the different climate conditions between the years.

Most of the previous studies pointed out that climate changes would cause spatiotemporal distribution changes of CH_4_ emissions from paddy fields. In the large-scale studies of China, most studies tended to concentrate on CH_4_ emissions from the rice paddies of the southern areas. As a result, CH_4_ emissions from paddy fields in northeast China have sometimes been neglected; few papers have studied the impacts of climate change on CH_4_ emissions through combining a global climate model (GCM) with different RCPs. Heilongjiang Province is located in the cold region of northeast China, and is sensitive to climate change [23], which will inevitably affect CH_4_ emissions from paddy fields in cold regions. The rice planting area in Heilongjiang Province of China has increased greatly since the 1990s; however, changes to the rice-planting areas in other provinces of China have been relatively small. According to statistics, the rice-planting area in Heilongjiang Province increased by 249% from 1990 to 2015, reaching 3.15 × 10^6^ ha [24,25]. Relevant studies have shown that the suitable planting boundary of rice in Heilongjiang Province will continue to expand northward under future climate change [26,27]. Therefore, with the gradual increase of the rice-planting area in Heilongjiang Province, the study of the impacts of climate change on spatiotemporal distributions of CH_4_ emissions from paddy fields in Heilongjiang Province need to be introduced as a matter of concern. 

To date, most of the studies on CH_4_ fluxes from paddy fields in the cold region of Heilongjiang Province have been site experiments. These experiments revealed the mechanism of CH_4_ fluxes from paddy fields in cold regions and provided reliable data support [28,29,30]. However, it was difficult for these site experiments to estimate CH_4_ emissions over a larger area, and the temporal and spatial variation of CH_4_ emissions from paddy fields in cold regions under climate change is still unclear. Therefore, the estimation of CH_4_ fluxes must rely on the model to expand the sample number of regional CH_4_ fluxes, and then assess the potential mitigation measures.

**Figure 1:**
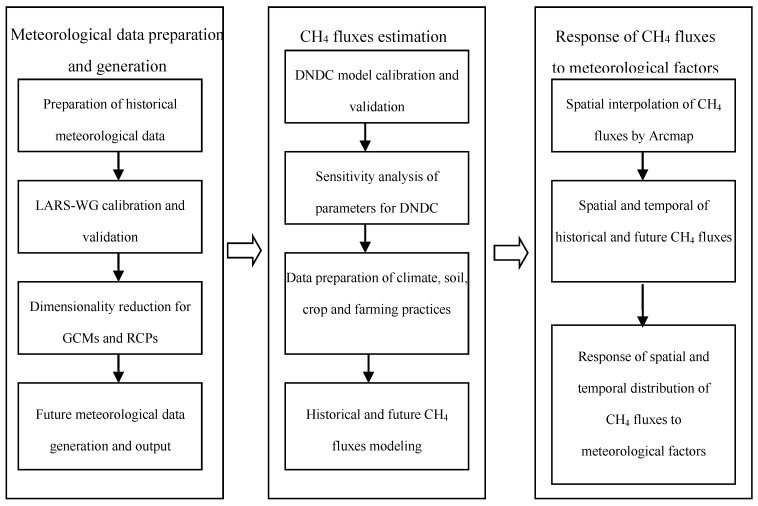
A systematic flow chart for estimating CH_4_ under climate change conditions.

In order to investigate the spatiotemporal distribution of CH_4_ fluxes in the rice paddies in Heilongjiang Province, firstly, the daily meteorological data of 26 stations in Heilongjiang Province from 1960 to 2015 were used to calibrate and validate the Long Ashton Research Station Weather Generator (LARS-WG) dimensionality reduction meteorological model and generate the climate data for 2021–2080 in Heilongjiang Province under RCP4.5 and RCP8.5. Secondly, the DNDC model was calibrated and validated by the CH_4_ fluxes data of a site experiment, and the sensitive factors affecting CH_4_ fluxes were analyzed by sensitivity analysis. Finally, the temporal and spatial distribution characteristics of CH_4_ fluxes in Heilongjiang Province in the past and future under different climate scenarios were simulated by the DNDC model, and the effects of climate factors on CH_4_ emission fluxes were discussed. The purpose of this study was to provide a basis for the establishment of a greenhouse gas emission inventory of paddy fields in cold regions. Figure 1 presents a system flow chart of this study.

## 2. Materials and Methods 

### 2.1. Overview of the Study Area

**Figure 2:**
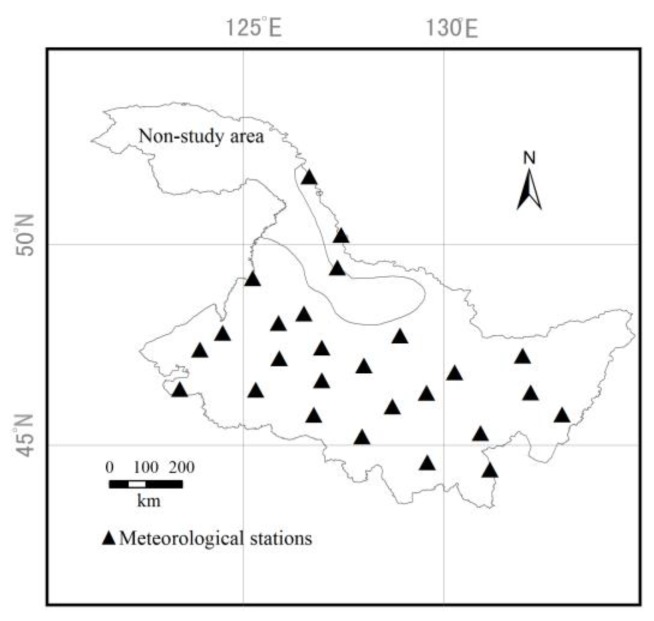
Study area and distribution of meteorological stations.

Heilongjiang Province is located in the northeast of China (121°11′–135°05′ E, 43°26′–53°33′ N), which is the highest-latitude province in China. It belongs to the continental monsoon climate in the cold and temperate zones. The frost-free period is relatively short. The crop growing season lasts from early May to early October. The total land area of Heilongjiang Province is 4.73 × 10^5^ ha, and the agricultural land area accounts for 83.5% of the total land area of Heilongjiang Province. The annual average temperature is 2.9 °C, the annual average rainfall is 526 mm, the soil is fertile, and the organic matter is high, which is conducive to the growth of crops. The sixth accumulated temperature zone in Heilongjiang Province is not suitable for rice cultivation; therefore, it is not the study area in this paper [31]. The study area of this paper is shown in Figure 2.

### 2.2. Future Climate Generation

#### 2.2.1. Introduction of LARS-WG Model

The LARS-WG (Long Ashton Research Station Weather Generator) is a random weather generator that can be used to simulate the historical and future meteorological data of a single station. It is a dimensionality reduction tool based on the GCM to generate local-scale climate scenarios. It uses the daily meteorological data of a given location to calculate a set of parameters for fitting probability distribution and their correlations. A synthetic weather time series of arbitrary length is generated by using the randomly selected values from the appropriate distribution [32]. The model has been validated in Europe, America, and Asia with reliable high performance [33]. The LARS-WG 6.0 used in this paper combines the Coupled Model Intercomparison Project 5 (CMIP5) in the IPCC Fifth Assessment Report for climate prediction. The process of weather generation is divided into the following three steps [32,33,34]:

(1) Model calibration: Statistical characteristics of historical meteorological parameters, including maximum temperature (Tmax), minimum temperature (Tmin), rainfall, and radiation (Rad) were analyzed through the "station analysis" function and stored in the corresponding parameter files.

(2) Model validation: The synthetic meteorological data with the same statistical characteristics as the historical meteorological data were generated by the parameter files of historical meteorological data in the process of model calibration. According to the comparison between the statistical characteristics of historical meteorological data and synthetic meteorological data, the simulation capability and applicability of the LARS-WG model were evaluated.

(3) Generation of synthetic meteorological data: The synthetic meteorological data corresponding to the GCM climate scenario are generated by using the parameter files in the calibration process of the model.

#### 2.2.2. Calibration of models and generation of future meteorological parameters

In this paper, the daily Tmax, Tmin, rainfall, and Rad of 26 meteorological stations (Figure 2) in Heilongjiang Province from 1960 to 2015 were used as historical input data for LARS-WG calibration and verification. The historical meteorological data was obtained from the China National Meteorological Information Center (http://data.cma.cn/). In order to evaluate the performance of LARS-WG, the Kolmogorov–Smirnov (K-S) test was performed on testing the equality of the seasonal distributions of dry and wet series (WDseries), distributions of daily rainfall (RainD), distributions of daily Tmax (TmaxD), distributions of daily Tmin (TminD), and distributions of daily Rad (RadD) calculated from the history data and downscale data, and a *t* test was performed on testing equality of the monthly mean rainfall (RainM), monthly mean of Tmax (TmaxM), monthly mean of Tmin (TminM), and monthly mean of Rad(RadM). For each station, the number of WDseries tests was eight, and the number of tests for other parameters was 12. The smaller the average number of meteorological station parameters *P* < 0.05, the better the performance of LARS-WG [35]. Table 1 shows that LARS-WG can well simulate the above parameters, and the model is more capable of simulating the monthly distribution of each parameter than the monthly mean of each parameter in the study area of this paper.

Climate data based on a GCM are widely regarded as the most acceptable model for studying climate change. Under different GCM scenarios, the magnitude of variation of meteorological factors is uncertain. Therefore, two RCPs under four GCMs in CMIP5 supported by LARS-WG 6.0 are selected to simulate future climate change. The main information of the four GCMs used is shown in Table 2 [36]. RCP 8.5 and RCP 4.5 represent radiation forcing values of 8.5 W/m^2^ and 4.5 W/m^2^ by 2100, respectively [37]. The meteorological data of two RCPs under the output of four GCMs by LARS-WG include daily Tmax, daily Tmin, daily rainfall, and daily Rad from 2021 to 2080.

### 2.3. CH_4_ Simulation

#### 2.3.1. Introduction of DNDC Model

The DNDC model is a geobiochemical model that is used to simulate greenhouse gas emissions from different agroecosystems. It has been widely used to simulate CH_4_ emissions from paddy fields all over the world over the past 20 years. Numerous studies have shown that DNDC can be used to estimate CH_4_ emissions from paddy fields at a regional scale [38,39,40,41]. The model also has a good simulation performance in the study area and near latitudes [22,40,42]. The model consists of two parts. The first part includes three sub-models: soil climate, crop growth, and soil organic matter decomposition. According to the input meteorological, soil, crop, and farming management data, the dynamic changes of soil temperature, water, redox potential, chemical substrate concentration, and other factors in soil environmental conditions are simulated. The second part includes three sub-models of nitrification, denitrification, and fermentation, which simulate the effects of various factors in soil on microbial activity and chemical reaction rate, and calculate greenhouse gas emissions [18]. The latest version of the DNDC model is DNDC95, which was used in this paper.

#### 2.3.2. Calibration and Validation of DNDC 

A field experiment was used to calibrate and validate the simulation capability of the DNDC model for CH_4_ emissions in this study area. The experiment was conducted in 2017 at the National Rice Irrigation Experiment Center in Suihua City, Heilongjiang Province (127°40′ E, 46°57′ N). The annual average temperature is 2~3 °C, the annual average rainfall is 500~600 mm, and the frost-free period is 128 days. The soil is loam. The bulk density of 0–10-cm soil is 1.26 g/cm^3^, the clay content is 21%, the porosity is 0.45%, the organic matter content is 41.8 g/kg, and the pH is 6.4. The rice variety was “Longqing 3” (*japonica*), which is the main local cultivar, with a planting density of 30 cm × 10 cm. It was transplanted on 20 May 2017 and harvested on 20 September 2017. Except for mid-season drainage from 1 July to 7 July 2017, the water layer was maintained in paddy fields during the rice growth period. The chemical fertilizers that were used were urea (N 46%), superphosphate (P_2_O_5_ 12%), and potassium chloride (K_2_O 60%), respectively. A total of 135 kg/hm^2^ of N, 45 kg/hm^2^ of P_2_O_5_, and 80 kg/hm^2^ of K_2_O were used. For N fertilizer, the ratio of types of fertilizer was 4.5:2:1.5:2 in the sequence of basal fertilizer: tillering fertilizer: promoting flower fertilizer: preserving flower fertilizer. K_2_O fertilizer was used twice as the basal fertilizer and 8.5 leaf age, respectively, with a ratio of 1:1. P_2_O_5_ fertilizer was applied once as the basal fertilizer. The maximum biomass values of grain, leaf, stem, and root were 4600 kg C/hm^2^, 1859 kg C/hm^2^, 1957 kg C/hm^2^, and 1370 kg C/hm^2^, respectively. The content of C and N in each part was determined by Element Analyzer (Flash 2000 HT, Thermo Fisher Science, USA), and the methods for measuring are described in reference [43] and [44]. The C/N ratios of grain, leaf, stem, and root adjusted by DNDC model parameters were 46, 58, 58, and 72, respectively. The nitrogen fixation index of local rice calculated by our research group was 1.26. The daily meteorological data in the year of the experiment were derived from the automatic weather station of the experiment station, including daily Tmax, Tmin, rainfall, average wind speed, average humidity, and radiation. For the observation and calculation methods of CH_4_ fluxes, refer to reference [30].

The above meteorological data, soil parameters, crop parameters, and farming measures parameters were input into the DNDC model, and the crop parameters were adjusted by the turn-and-just method. The model was calibrated and validated by comparing the measured CH_4_ fluxes and the simulated CH_4_ fluxes. A determinant coefficient (R^2^), root mean square error (RMSE), and model efficiency coefficient (EF) were used to evaluate the “goodness of fit” of the DNDC model for CH_4_ flux simulation. The root mean square error (RMSE) is used to measure the consistency between the simulated and observed CH_4_ fluxes. The EF value is less than or equal to one; a positive value indicates that the simulated values describe the trend in the measured data better than the mean of the observed values [45]. The calculation formulas are as follows:

(1) $$RMSE=\sqrt{\frac{(F_{s}-F_{o}{)}^{2}}{n}}$$

(2) $$EF=\frac{\sum_{i=1}^{n}(O_{i}-\bar{O}{)}^{2}-\sum_{i=1}^{n}{\left(S_{i}-O_{i}\right)}^{2}}{\sum_{i=1}^{n}(O_{i}-\bar{O}{)}^{2}}$$

where, *F_s_* and *F_o_* in Equation (1) denotes the simulated and observed CH_4_ fluxes in the site experiment, and *n* is the total number of CH_4_ observations. In Equation (2), *O_i_* are the observed CH_4_ fluxes, *S_i_* are the simulated CH_4_ fluxes, and $\bar{O}$ is the mean of the observed CH_4_ fluxes.

#### 2.3.3. Calibration and Validation of DNDC Sensitivity Analysis

CH_4_ emissions from paddy fields are affected by many factors, some of which may be more sensitive than others [46]. Sensitivity analysis help to better understand the sensitivity of the model to various parameters and provide support for the calibration and validation. Therefore, sensitivity analysis of the DNDC model was performed to evaluate the response of simulation results to input parameters [18,22]. Climate, soil, and crop parameters of the site experiment were taken as the initial conditions for sensitivity analysis, and a single input parameter was changed within the default range of the DNDC model system, while all the other input parameters remained unchanged. Each input parameter was run 500 times to ensure the reliability of the results. For those more sensitive factors, attention should be paid during the model simulation to improve the reliability of the data.

#### 2.3.4. Constructing Regional Simulation

The DNDC model needs meteorological data, soil data, crop parameters, and farmland management measures to simulate CH_4_ fluxes from paddy fields. In this study, the annual CH_4_ fluxes from 26 meteorological stations in the study area were simulated by the DNDC model. For CH_4_ fluxes from 2021 to 2080, the average values of four GCMs under two RCPs were calculated respectively, and then, the spatial and temporal variations of CH_4_ fluxes were expressed by spatial interpolation. The period 1960–2015 was divided into three periods: 1960–1979 (1970s), 1980–1999 (1990s), and 2000–2015 (2010s). The period 2021–2080 was divided into 2021–2040 (2030s), 2041–2060 (2050s), and 2061–2080 (2070s).

For the simulation of the period 1960–2015, the daily meteorological data of 26 meteorological stations in Heilongjiang Province from 1960 to 2015 were selected, including daily Tmax, Tmin, average relative humidity, average wind speed, and rainfall. For the simulation of 2021–2080, the daily meteorological data of 26 meteorological stations were output by LARS-WG, including daily Tmax, Tmin, rainfall, and Rad. Considering the change of CO_2_ concentration under climate change, the background CO_2_ concentration in 1960 was 317 ppm and the growth rate was 1.58 ppm/a. For future climate simulation, the atmospheric CO_2_ background concentrations were 411 ppm and 415 ppm under RCP 4.5 and RCP 8.5 in 2021, respectively, and 531 ppm and 758 ppm in 2080, respectively [37].

The soil physical and chemical properties parameters at soil depths of 0 to 30-cm input for the simulation were derived from the HWSD (http://www.fao.org/land-water/databases-and-software/hwsd/en/), including: soil type, bulk density, clay content, organic matter content, porosity, and pH value. Paddy cultivation in different regions of the study area were derived from the “*Compilation of Paddy Field Irrigation Experiment Data in Heilongjiang Province*”, which included the date of paddy field preparation and paddy puddling, transplanting, mid-season drainage, fertilization regime, and harvest date of 30 experiment stations in 542 experimental years from 1952 to 2002. In order to reduce the uncertainty of the simulation results, the following assumptions were made in this study. (1) Transplanting was used for rice planting. The cultivation techniques of rice seedlings nurseries in upland bed in Heilongjiang Province began in the 1950s, and were well promoted with the development of plastic film technology in the 1960s and 1970s. This technology is in line with the cold rice cultivation environment and rice growth physiology. (2) There was no change in rice transplanting and harvesting time. Due to seedlings nursery and upland bed cultivation technology, the rice transplanting date was more flexible and less affected by temperature. Farmers in different regions depended on experience to determine fixed transplanting and harvesting dates. (3) The stubble remained 10% after harvest. In the historical period, the straw harvested in the study area was used for living fuel and animal feed. Currently, crop straw burning is prohibited in Heilongjiang Province. In the future, straw will be developed into new energy, decorative materials and other uses. (4) Setting up mid-season drainage. This technology has been widely used by farmers in the study area because it reduces the ineffective tillering of rice, improves the fertilizer utilization rate, improves soil aeration, and effectively reduces CH_4_ emissions [46]. (5) There was no change in rice varieties during the study period.

#### 2.3.5. Climatic Tendency

The climatic tendency of CH_4_ fluxes is expressed by a linear equation by using the least square method. The formulas is as follows:

(3) $${\hat{Y}}_{i}=at+b$$

where ${\hat{Y}}_{i}$ is the fitting value of CH_4_ fluxes, *t* is the corresponding year, and *a* and *b* are the regression coefficients.

*a* × 10 is the climatic tendency, which represents the change rate of CH_4_ fluxes every 10 years (10a). The positive value indicates an increasing trend, while the negative value indicates a decreasing trend.

### 2.4. Data Processing

To meet the requirements of the input and output meteorological format of the DNDC model and the LARS-WG model, the meteorological data format was processed by MATLAB 2004b. The CH_4_ fluxes and their climatic tendencies were interpolated and mapped by using the spatial analysis function of the Arcmap 10.2 toolbox.

## 3. Results

### 3.1. Calibration and Validation of DNDC and Sensitivity Analysis

#### 3.1.1. Calibration and Validation of DNDC for CH_4_ Fluxes

**Figure 3:**
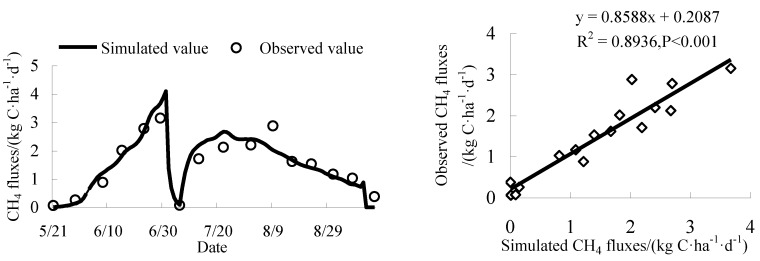
Comparison of simulated and observed CH_4_ fluxes in the validated experiment.

Comparisons of the CH_4_ fluxes observed in the site experiment with those simulated by DNDC are shown in Figure 3. Except for individual differences, the simulation results of DNDC are in good agreement with the observed results. Regression analysis shows that the regression coefficient R^2^ is 0.89, the slope of regression equation is close to one, and the intercept is relatively small. The correlation between simulated and observed values was very high (*P* < 0.001). The RMSE value and EF value are 0.35 and 0.87, respectively. The RMSE value is relatively small, and the efficiency coefficient of the model is high, which indicates that the simulation value can well reflect the actual change rule of CH_4_ fluxes. In conclusion, DNDC can well simulate and predict the seasonal variation of CH_4_ fluxes in this study area.

#### 3.1.2. Sensitivity Analysis of Factors to CH_4_ Fluxes

**Figure 4:**
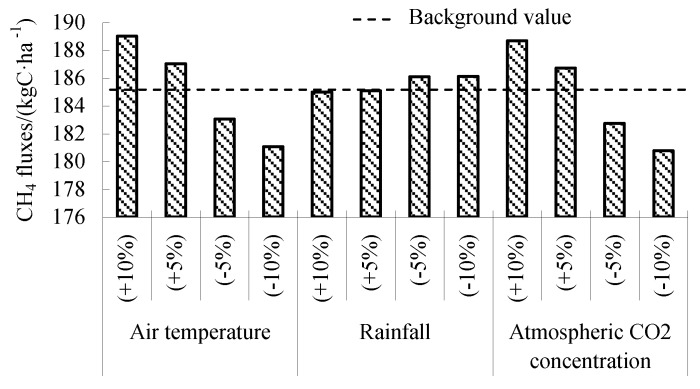
Sensitive analysis of meteorological parameters on driving CH_4_ flux in validated experiments.

The response of CH_4_ fluxes to climate factors was studied by fluctuating 10% of the air temperature, rainfall, and atmospheric CO_2_ concentration. As shown in Figure 4, higher air temperature and higher atmospheric CO_2_ concentration promote the increase of CH_4_ fluxes. This may be because the increase of air temperature is beneficial to the decomposition of soil organic matter and the activity of methanogens [7]. Increased atmospheric CO_2_ concentration provides more metabolic substrates for methane production [9,10]. The change of annual rainfall has little effect on CH_4_ fluxes. With the decrease of rainfall, the CH_4_ flux increases slightly.

**Figure 5:**
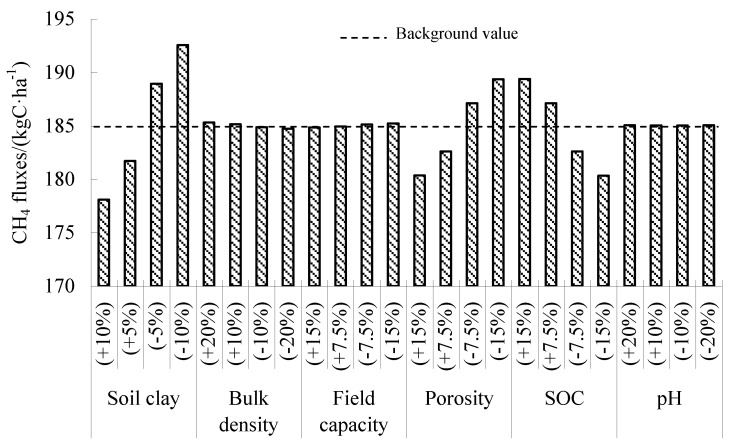
Sensitive analysis of soil parameters on driving CH_4_ flux in validated experiments.

A sensitivity analysis of soil parameters showed that soil clay content was the most sensitive factor affecting CH_4_ fluxes, followed by soil porosity and SOC (Figure 5). Soil with high clay content limits CH_4_ fluxes due to the adsorption of clay, which limits the use of dissolved organic carbon by microorganisms. The increase of porosity will promote the oxidation of methanogens to produce CH_4_, and SOC will provide more substrates for methanogens to produce CH_4_. Since paddy fields are flooded most of the time during the rice-growing period, the change of soil moisture and dryness is small, and the soil condition is relatively stable; therefore, the effects of soil bulk density, field water capacity, and pH on CH_4_ fluxes are small [22].

**Figure 6:**
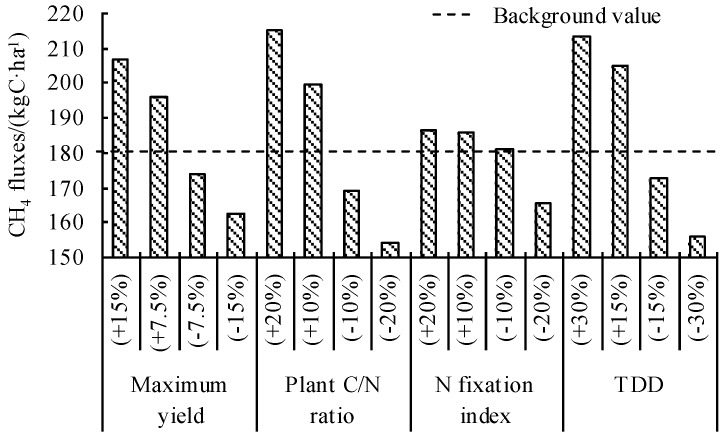
Sensitive analysis of crop parameters on driving CH_4_ flux in validated experiments where TDD is thermal degree days for maturity.

A sensitivity analysis of crop parameters showed that CH_4_ fluxes were more sensitive to maximum yield, plant C/N ratio, and thermal degree days for maturity (TDD), but less sensitive to the N fixation index (Figure 6). With the increase of the above parameters, CH_4_ fluxes showed an increasing trend. This is because with the increase of TDD, more dry matter can be accumulated in crops with the same proportion of biomass distribution in different parts of the plant. Increasing the maximum yield also increases biomass accumulation in plants, since it is beneficial for increasing plant droppings and root exudates, which provides more carbon sources for CH_4_ production. Increasing the plant C/N ratio also provides more carbon sources for methanogens.

### 3.2. Temporal and Spatial Distribution Characteristics of CH_4_ Fluxes

#### 3.2.1. Climate Change During the Study Period

The historical and future changes of climate parameters in the study area are shown in Table 3. During the 1970s–2010s, the daily Tmin, daily Tmax, annual rainfall, and atmospheric CO_2_ concentration showed an increasing trend, while the annual Rad showed a decreasing trend. During the 2030s–2070s, the predicted daily Tmin, daily Tmax, annual rainfall, and atmospheric CO_2_ concentration continued to increase, and the increase of four meteorological parameters under RCP 8.5 was greater than that under RCP 4.5. However, the annual Rad continued to decrease, and the reduction under RCP 8.5 was greater than that under RCP 4.5.

#### 3.2.2. Temporal and Spatial Distribution Characteristics of CH_4_ Fluxes under Historical Climate.

**Figure 7:**
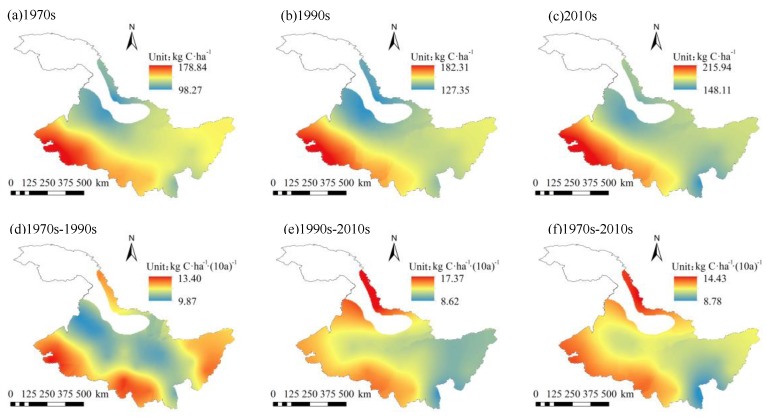
Spatial distributions of CH_4_ fluxes in (**a**) 1970s, (**b**) 1990s, and (**c**) 2010s and spatial distributions of CH_4_ fluxes climatic tendency during (**d**) 1970s–1990s, (**e**) 1990s–2010s, and (**f**) 1970s–2010s.

**Figure 8:**
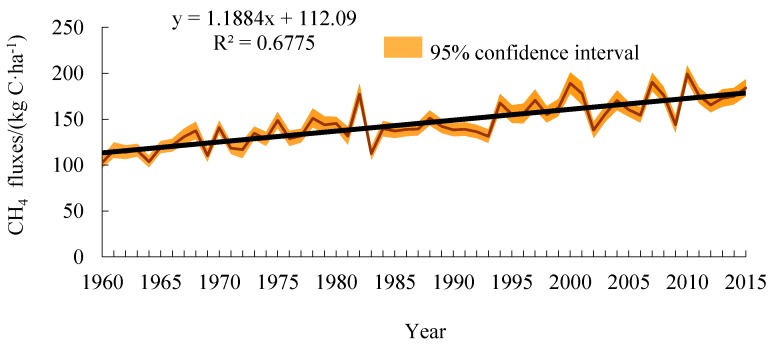
Changing tendency of average CH_4_ fluxes of meteorological stations during 1960–2015.

The spatial distribution of CH_4_ fluxes and its climatic tendency in the study area from 1960 to 2015 is shown in Figure 7. CH_4_ fluxes ranged from 98.27 kg C/ha to 215.94 kg C/ha, with an average of 145.56 kg C/ha. CH_4_ fluxes in the 1970s, 1990s, and 2010s were 125.80 kg C/ha, 146.42 kg C/ha, and 170.57 kg C/ha, respectively. CH_4_ emission fluxes showed a decreasing trend from west to east. The high-value areas were mainly distributed in the western part with an average of more than 175 kg C/ha. The low-value areas were mainly distributes in the northern area, with an annual average value of less than 120 kg C/ha. The climatic tendency was between 8.78–14.43 kg C/ha/(10a) with an average of 11.88 kg C/ha/(10a). The average CH_4_ fluxes from 26 meteorological stations in the study area showed a fluctuating and increasing trend (Figure 8). Among them, the northern and western parts increased at a higher rate, with a climatic tendency rate of more than 14 kg C/ha/(10a).

From the 1970s to the 1990s, the CH_4_ fluxes showed an increasing trend. The climatic tendency was between 9.87–13.40 kg C/ha/(10a), with an average of 11.42 kg C/ha/(10a). Among them, the climatic tendency in the central part was relatively low, while the climatic tendency in the eastern, southern, and western regions were relatively high. From the 1990s to the 2010s, the average climatic tendency of CH_4_ fluxes was slightly higher than that of the 1970s–1990s, and the climatic tendency was between 8.62–17.37 kg C/ha/(10a), with an average of 12.62 kg C/ha/(10a). In the eastern region, the increasing rate slowed down, while in the northern part, the increasing rate increased dramatically. The climatic tendency of the northern part was greater than 17 kg C/ha/(10a).

#### 3.2.3. Spatial and Temporal Distribution Characteristics of Methane Emission Fluxes in Future Climate

**Figure 9:**
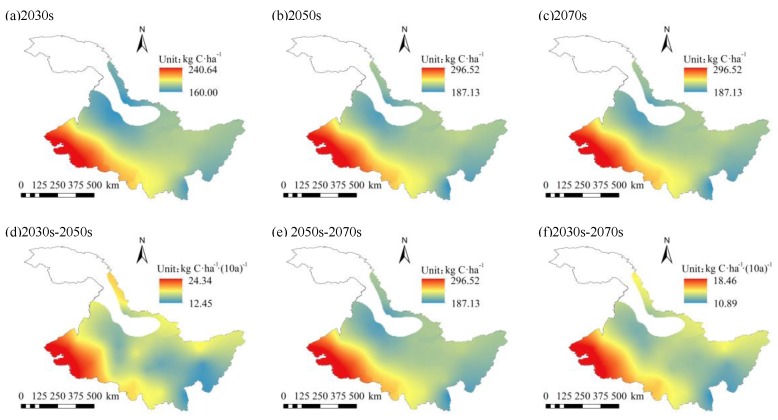
Spatial distributions of CH_4_ fluxes in the (**a**) 2030s, (**b**) 2050s, and (**c**) 2070s, and spatial distributions of CH_4_ fluxes climatic tendency during (**d**) 2030s–2050s, (**e**) 2050s–2070s, and (**f**) 2030s–2070s under representative concentration path (RCP) 4.5.

**Figure 10:**
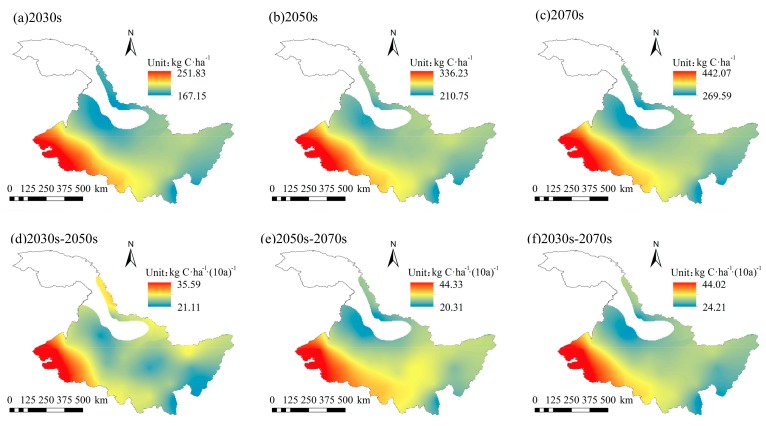
Spatial distributions of CH_4_ fluxes in (**a**) 2030s, (**b**) 2050s, and (**c**) 2070s, and spatial distributions of CH_4_ fluxes’ climatic tendency during (**d**) 2030s–2050s, (**e**) 2050s–2070s, and (**f**) 2030s–2070s under RCP 8.5.

Taking the mean of CH_4_ fluxes from 1960 to 2015 as the background value of historical CH_4_ fluxes, the temporal and spatial variation characteristics of CH_4_ fluxes in the study area under future climate change were studied. From Figure 9 and Figure 10, it can be seen that under RCP 4.5 and RCP 8.5, CH_4_ fluxes ranged from 160.00 to 318.68 kg C/ha and 167.15 to 442.07 kg C/ha between 2021–2080, with average values of 213.46 kg C/ha and 252.19 kg C/ha, respectively. Compared with historical background values, CH_4_ fluxes increased by 67.50 kg C/ha and 106.23 kg C/ha between 2021–2080 under RCP 4.5 and RCP 8.5, respectively. The average CH_4_ flux under RCP 8.5 increased more than that under RCP 4.5. The spatial distribution of CH_4_ fluxes under RCP 4.5 and RCP 8.5 between the future was similar to the historical distribution of CH_4_ fluxes, and it tended to decrease from west to east. However, under RCP 4.5 and RCP 8.5, the climatic tendency of CH_4_ fluxes between 2021–2080 ranged from 10.89 to 18.46 kg C/ha/(10a) and 24.21 to 44.02 kg C/ha/(10a), respectively. The average climatic tendencies were 13.40 kg C/ha/(10a) and 29.86 kg C/ha/(10a), respectively. Compared with the historical climatic tendency, the climatic tendency of CH_4_ fluxes increased by 1.52 kg C/ha/(10a) and 17.98 kg C/ha/(10a) respectively between 2021–2080 under RCP 4.5 and RCP 8.5. The climatic tendency of CH_4_ fluxes under RCP 8.5 was higher than that under RCP 4.5. The spatial distribution of the climatic tendency of CH_4_ fluxes was different from that between 1960–2015. The climatic tendency of CH_4_ fluxes decreased from west to east between 2021–2080. The high-value area was mainly distributed in the west, while the low-value area was mainly distributed in the east.

**Figure 11:**
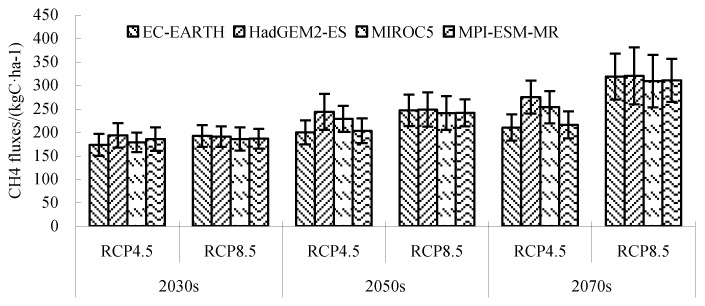
Simulated CH_4_ fluxes of each global climate model (GCM) under RCP 4.5 and RCP 8.5.

Under RCP 4.5, the CH_4_ fluxes of each GCM in different periods were quite different, while the difference of CH_4_ fluxes of each GCM in different periods was small under RCP 8.5 (Figure 11). Under RCP 4.5 and RCP 8.5, the climatic tendency of CH_4_ fluxes in the 2030s–2050s ranged from 12.45 kg C/ha/(10a) to 24.34 kg C/ha/(10a) and 21.11 kg C/ha/(10a) to 35.59 kg C/ha/(10a), with an average of 16.34 kg C/ha/(10a) and 24.09 kg C/ha/(10a), respectively. The climatic tendency distribution of CH_4_ fluxes under RCP 4.5 and RCP 8.5 was similar. However, the climatic tendency under RCP 8.5 was higher than that under RCP 4.5. In the 2050s–2070s period, the climatic tendency of CH_4_ fluxes under RCP 4.5 and RCP 8.5 ranged from 7.31 kg C/ha/(10a) to 10.51 kg C/ha/(10a) and 20.31 kg C/ha/(10a) to 44.33 kg C/ha/(10a), with an average of 8.61 kg C/ha/(10a) and 28.86 kg C/ha/(10a), respectively. Compared with the 2030s–2050s stage, the climatic tendency under RCP 8.5 continued to increase spatially and temporally, with a big increase in the central part, while the increase in the northern part was smaller. However, the climatic tendency under RCP 4.5 tended to decrease, and was even less than the climatic tendency of CH_4_ fluxes in the historical period. The decreases in the north and west parts were larger. Overall, the spatial distribution of CH_4_ fluxes in the 2030s, 2050s, and 2070s stages under RCP 4.5 and RCP 8.5 showed a decreasing trend from west to east temporally, and showed a continuous increasing trend spatially. The climatic tendency of CH_4_ fluxes varied greatly in different periods. Under RCP 4.5, the climate tendency of CH_4_ flux decreased, while under RCP 8.5, the climate tendency increased.

### 3.3. Effects of Climate Factors on CH_4_ Fluxes

**Figure 12:**
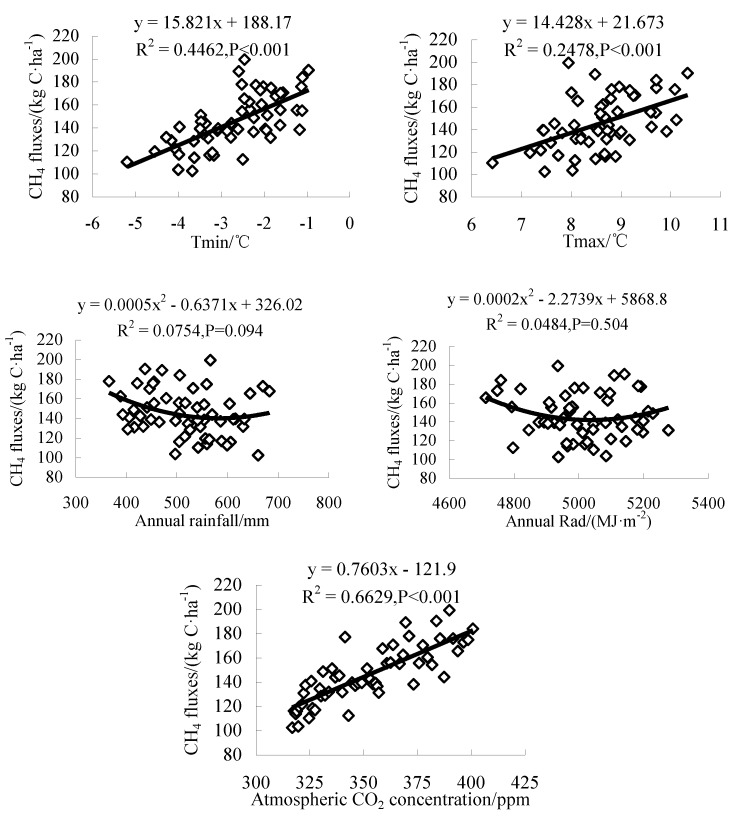
Regression analysis between meteorological parameters and CH_4_ fluxes.

As the uncertainty of future meteorological data is high, in order to study the effects of climate factors on CH_4_ fluxes, the correlation analysis and regression analysis of historical meteorological data and CH_4_ emission fluxes at each station in the study area were carried out. From Figure 12, CH_4_ fluxes increased with the increase of Tmax, Tmin, and atmospheric CO_2_ concentration, and were significantly correlated with them (*P* < 0.001). The CH_4_ fluxes decreased first and then increased with the increase of rainfall and Rad, and the correlation between them was not significant. Therefore, the increase of Tmax, Tmin, and atmospheric CO_2_ concentration at each station in the study area from 1960 to 2015 (Table 3) promoted the increase of CH_4_ fluxes. Compared with historical average, the Tmin, Tmax, and the atmospheric CO_2_ concentration in the 2070s period increased by 1.90 °C, 2.17 °C, and 92.80 ppm (Table 3), respectively, resulting in a 92.8 kg C/ha increase in CH_4_ fluxes, which was an increase of 64%. Under RCP 8.5 in the 2070s, the Tmin, Tmax, and atmospheric CO_2_ concentration increased by 3.71 °C, 3.86 °C, and 172.69 ppm (Table 3), respectively, resulting in an increase of 172.69 kg C/ha in CH_4_ fluxes, with an increase of 118%, which was greater than that under RCP 4.5. The above analysis showed that increasing temperature and atmospheric CO_2_ concentration can accelerate CH_4_ fluxes.

**Figure 13:**
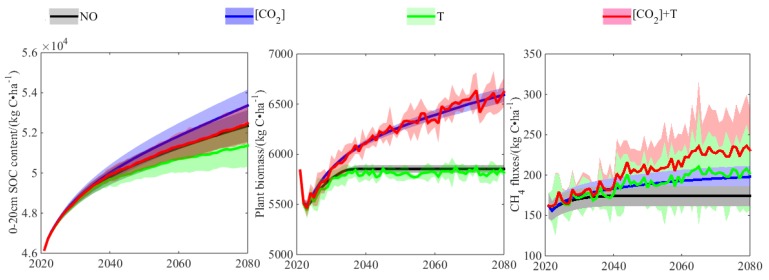
Response of SOC content between 0–20 cm, plant biomass, and CH_4_ fluxes to the changing climate condition under RCP 4.5; the solid line represents the mean among the four GCMs, while the shaded area represents the corresponding 95% CI. Scenarios: NO, no change in atmospheric CO_2_ concentration [CO_2_] and air temperature (T); [CO_2_] change in [CO_2_] alone; T, change in T alone; [CO_2_]+T, both changes in [CO_2_] and T. See Section 3.3 for detail.

In order to further study the effects of atmospheric CO_2_ concentration and temperature on CH_4_ fluxes, the four simulation scenarios are as follows: (1) the atmospheric CO_2_ concentration and temperature do not change; (2) the atmospheric CO_2_ concentration increases, but the temperature does not change; (3) the atmospheric CO_2_ concentration does not change, but the temperature changes; and (4) both the atmospheric CO_2_ concentration and temperature change. Figure 13 showed the response of SOC content in the 0–20-cm soil layer, biomass (including leaf, stem, and root), and CH_4_ fluxes to climate changes under RCP 4.5 in the site experiment. Increased atmospheric CO_2_ concentration and successive years of stubble returning would lead to the increase of SOC content in the 0–20-cm soil layer and the accumulation of plant biomass year by year. The decomposition of SOC would be promoted by the increase of air temperature, and the plant biomass will be increased in the coming 20 years. Compared with scenario one, the CH_4_ fluxes in scenario two, scenario three, and scenario four increased by 13.74%, 16.31%, and 32.78% at the end of the simulation. CO_2_ enhanced the root system of crops through plant photosynthesis [47], provided more metabolic substrates for CH_4_ production, and increased the aerenchyma of plants by increasing the biomass, thus providing a channel for CH_4_ emissions [48]. Previous studies have shown that CH_4_ fluxes had a significant correlation with the biomass of crops [49]. Increasing soil temperature would promote the decomposition of SOC and increase the activity and amount of methanogens. Compared with CH_4_ production, the CH_4_ oxidation process is less affected by temperature [50]. Under future climate scenarios, atmospheric CO_2_ concentration and temperature will affect the CH_4_ fluxes of paddy field through the joint effects of soil and plant.

## 4. Discussion

### 4.1. Comparisons with Previous Studies

In this paper, the temporal and spatial distribution of CH_4_ fluxes from paddy fields in cold regions of Heilongjiang Province under historical and future conditions were studied. The high-latitude record of CH_4_ fluxes from paddy fields simulated by the DNDC model was refreshed. The dynamic process of CH_4_ fluxes from paddy fields in this region was well simulated by the calibration and validation of the site experiment. Compared with previous studies, some similarities and differences exist. Zhang et al. [7,51] studied the CH_4_ flux dynamics of Sanjiang Plain in eastern Heilongjiang Province from 1990 to 2010 by the DNDC model. Sensitivity analysis showed that CH_4_ fluxes were more sensitive to temperature, soil clay content, and SOC, but less sensitive to rainfall, soil bulk density, and pH, which was consistent with the results of this study, but he did not carry out a sensitivity analysis on crop parameters. His research results showed that the average CH_4_ fluxes in 1990, 2000, and 2010 were 71 kg C/ha, 137 kg C/ha, and 180 kg C/ha, respectively. In this study, the average CH_4_ fluxes in 1990, 2000, and 2010 were 138 kg C/ha, 189 kg C/ha, and 199 kg C/ha, respectively, which were higher than the results of the previous study. The main reason may be due to the difference of the site experiment results for DNDC model validation. The average CH_4_ flux of the site experiment used in this study was larger than that of his study. Therefore, in regional simulation, the calibration and validation of the model has a greater effect on the simulation results. In future regional simulation, multi-site experiments should be carried out in order to calibrate and validate the model and thus increase the reliability of the simulation. The results from another paper of Zhang’s showed that CH_4_ flux from paddy fields in Sanjiang Plain is mostly concentrated in the range of 50 to 600 kg C/ha, which is larger than that in this study area. The main reason is that the soil database used in this study is different from that in this paper. The spatial distribution of simulation results is quite different when using grid data to construct regional simulation. Zhang et al. [21] pointed out that CH_4_ fluxes under A1B and B1 scenarios in the three northeastern provinces from China (including, Heilongjiang Province, Jilin Province, and Liaoning Province) would increase from 160 kg C/ha to 200 kg C/ha between 2010–2050, which is similar to the change of CH_4_ fluxes under RCP 4.5 in this paper. The 2006 IPCC Guidelines for National Greenhouse Gas Inventories recommended a CH_4_ baseline emission factor of 1.3 kg CH_4_/ha/d for estimating CH_4_ emissions from paddy fields in regions or countries [46,52]. The growth period of rice in Heilongjiang Province is about 115 days, so the calculated CH_4_ fluxes is 112.13 kg C/ha, which is less than the results of CH_4_ fluxes at all of the historical periods in this study. This is mainly because the SOC in the black soil in Heilongjiang Province is higher compared with other regions, which provided a greater carbon source for CH_4_ production. In this study, many ecological driving factors affecting greenhouse gas emissions from paddy fields, such as climate, soil, and farmland management measures, were considered. Therefore, compared with the method recommended by the IPCC, the method adopted in this study has greatly improved the estimation of regional CH_4_ fluxes. There are few studies of CH_4_ emissions from paddy fields in Heilongjiang Province under future climate conditions. This study pointed out that CH_4_ fluxes and the climatic tendency of northern Heilongjiang Province under RCP 4.5 and RCP 8.5 conditions were relatively small. With the increase of accumulated temperature in the study area under climate change, the appropriate development of rice cultivation in this area would have better CH_4_ emission reduction advantages than other areas.

### 4.2. Effects of Climate Factors on CH_4_ Emissions

This study pointed out that the changes of atmospheric CO_2_ concentration and temperature were the main climatic factors affecting CH_4_ fluxes from paddy fields in cold regions. Tokida et al. [53] conducted a two-year experiment in Iwate Prefecture, Japan (39°38′ N). The effects of increasing atmospheric CO_2_ concentration (background concentration +200 ppm) and soil temperature (background temperature +2 °C) on CH_4_ emissions from paddy fields were studied. This was similar to the atmospheric CO_2_ concentration at RCP 4.5 and the amplitude of elevated temperature at the end of the simulation in this study. The atmospheric CO_2_ concentration, Tmin, and Tmax in the 2070s stage increased by 1.90 °C, 2.17 °C, and 172 ppm, respectively (Table 3). His results showed that increasing atmospheric CO_2_ concentrations, soil temperatures, and their combination increased CH_4_ fluxes by 26%, 44%, and 88%, respectively, which were larger values than the increase of 13.74%, 16.31%, and 32.78% in scenarios two, three, and four in this study under RCP 4.5, respectively. The climate conditions in his experimental area were similar to those in this study, but the latitude was lower than that in this study area. Higher temperatures are more conducive to CH_4_ fluxes. In his study, soil and surface water were heated directly to increase temperature, which is different from the temperature increase effect of 60 years that was used in this study. Under actual global warming conditions, soil temperature increases gradually, and SOC decreases gradually without the artificial addition of carbon sources. However, the initial soil condition of each treatment in his study was the same; therefore, the CH_4_ fluxes might be overestimated. In addition, although the temperature of the soil increased the biomass of the aboveground part, it accelerated the senescence of the root system, and reduced the biomass of the root system by 8%. This may provide more substrates for the production of CH_4_. Due to the heating method, the mid-season drainage was also not set in his experiment, which further overestimated CH_4_ fluxes. Tokida’s results and previous studies also pointed out that increasing CO_2_ concentration increased the plant biomass, while CH_4_ fluxes were positively correlated with both, and increased CH_4_ fluxes. Van et al. [3] pointed out through a meta-analysis that the increase of CO_2_ concentration (range 463 to 780 ppm) resulted in an increase of 43.4% in CH_4_ fluxes from paddy fields. The results of this study are similar to those of previous studies. Kazumori et al. [27] used an improved DNDC-Rice model to analyze the effects of RCP 4.5 on SOC content in a 30-cm soil layer, the biomass of the aboveground part, and the CH_4_ fluxes in double-cropping paddy fields in Suphan Buri, Thailand, from 2000 to 2060. The results showed that increasing atmospheric CO_2_ concentration alone could increase soil SOC content, biomass, and CH_4_ fluxes, which were the same as the results of this study. However, in his study, the increase of temperature did not improve the soil SOC content and the biomass of the aboveground parts, which was different from the results of this study. It may be that the tropical climate of Thailand is more conducive to the decomposition of soil SOC. Meanwhile, the soil in this study is frozen or thawed for most of the time except for the crop-growing season, which is conducive to the accumulation of soil SOC. Furthermore, a high temperature will produce temperature stress on rice plants, which is not conducive to crop growth, leading to a decline in the biomass above ground. Neither this study nor Kazumori’s studies considered the effects of crop varieties on CH_4_ fluxes. The study results showed that the rice cropping system in Heilongjiang Province has shown its adaptability to climate change, and the planting boundary tends to expand northward [26]. Later-maturing varieties gradually replace early-maturing varieties, which will inevitably increase the biomass of the aboveground parts and roots of rice, and increase the plant litter, stubble incorporated and the root exudates of rice plants. As DNDC sets the biomass and yield response with the increase of atmospheric CO_2_ concentration but not with the increase of temperature, the simulation results of CH_4_ fluxes in this study may be larger for historical periods, but smaller for future periods, resulting in the overall low climatic tendency for CH_4_ fluxes. Due to the climate, soil, and crop differences, the CH_4_ flux response on atmospheric CO_2_ concentration and temperature across different regions is not consistent.

### 4.3. Uncertainty and Prospect

There are many uncertainties in the simulation of CH_4_ fluxes in this study. For example, in the construction of regional simulation, although the meteorological, soil, crop parameters, and agricultural management measures adopted by 26 meteorological stations were representative in the local area, the distributions of relevant parameters between the meteorological stations were still very uncertain. The related parameters would also change with the effect of climate change. For example, this paper adopted the management system of rice experiment stations, but in the actual agricultural production, the management system was more flexible. In the years of low temperature, the mid-season drainage was not carried out to ensure the tiller number of rice. This increased CH_4_ fluxes. With the increase of accumulated temperature in different regions, rice varieties were constantly renewing, and the change of rice varieties would also lead to the difference of CH_4_ fluxes [54]. This study simulated the CH_4_ fluxes according to a mid-season drainage condition, without considering effect of irrigation pattern change. Zhang et al. [21] pointed out that CH_4_ fluxes decreased from 144 kg C/ha to 99 kg C/ha from 1960 to 1970 due to the large-scale popularization of mid-season drainage technology in rice-growing areas in China, and then increased due to the effects of yield and organic fertilizer. In addition, the deficiencies of the model may also lead to differences in the simulation results. As we mentioned above, DNDC does not set parameters for the adaptability of crop biomass with the increase of accumulated temperature, which may lead to differences in estimating CH_4_ fluxes. This paper pointed out that the cold black soil region under climate change would become an important CH_4_ emission source in China’s agricultural system. With the increase of the rice area in Heilongjiang Province, more site experiments are needed to provide a theoretical basis and data support for the regional estimation of CH_4_ fluxes, and also calibrate and validate the model to improve the accuracy of the estimation of regional CH_4_ fluxes.

## 5. Conclusions

This study simulated the historical and future spatiotemporal dynamics of CH_4_ from paddy fields in the cold region of Heilongjiang Province under different climate scenarios using a combination of the DNDC model and the LARS-WG model. The simulation results showed that from 1960 to 2015, the average CH_4_ flux in the study area was 145.56 kg C/ha, showing a decreasing trend from west to east. The average climate tendency was 11.88 kg C/ha/(10a). The climate tendencies of the CH_4_ fluxes in the northern and western parts were higher. Under RCP 4.5 and RCP 8.5, the average CH_4_ fluxes from 2021 to 2080 increased by 67.50 kg C/ha and 106.23 kg C/ha, respectively, compared with the average historical value. The spatial distribution tended to decrease from west to east. The average climatic tendency increased by 1.52 kg C/ha/(10a) and 17.98 kg C/ha/(10a), respectively, compared with the historical background values. The CH_4_ fluxes’ climate tendency decreased from west to east. The correlation analysis and regression analysis showed that CH_4_ fluxes increased with the increase of Tmin, Tmax, and atmospheric CO_2_ concentration (*P* < 0.001). Under future climate change scenarios, atmospheric CO_2_ concentration and temperature will affect the CH_4_ fluxes of paddy fields through the interaction of soil and plant in the cold region of Heilongjiang Province. To reduce the effects of atmospheric CO_2_ concentration and temperature on increasing CH_4_ fluxes, more attention should be paid to improve rice cultivars with low plant biomass to reduce CH_4_ and carbon substrate supply. Moreover, reducing the additional carbon source such as stubble and straw incorporation to control the increase of SOC should be also considered. Furthermore, effective and efficient policies and measures should be applied to technologies such as paddy water management and soil cultivation, as well as the training and education to the farmers to decrease the impact of climate change on CH_4_ fluxes [5,55].

## Figures and Tables

**Table 1:** Statistical tests comparing the historical meteorological data with synthetic date generated by the Long Ashton Research Station Weather Generator (LARS-WG) 6.0. Values signify seasonal distributions of dry and wet series (WDseries), distributions of daily rainfall (RainD), distributions of daily Tmax (TmaxD), distributions of daily Tmin (TminD), distributions of daily Rad (RadD), monthly mean rainfall (RainM), monthly mean of Tmax (TmaxM), monthly mean of Tmin (TminM), and monthly mean of Rad (RadM).

Parameters	WD Series	RainD	TminD	TmaxD	RadD	RainM	TminM	TmaxM	RadM
**The average number of** **each parameter (*P* < 0.05)**	**0.35**	**0.35**	**0.42**	**0.73**	**0.08**	**0.31**	**0.92**	**1.12**	1.96
Total tests	8	12	12	12	12	12	12	12	12

**Table 2:** Four global climate models (GCMs) selected for LARS-WG simulation in this study.

GCMs	Research Center	Countries and Regions	Grid Resolution
EC-EARTH	EC: Earth consortium	Europe	1.125° × 1.125°
HadGEM2-ES	United Kingdom (UK) Meteorological Office	UK	1.25° × 1.88°
MIROC5	University of Tokyo, National Institute for Environmental	Japan	1.39° × 1.41°
MPI-ESM-MR	Max Planck Institute for Meteorology	Germany	1.85° × 1.88°

**Table 3:** Changes of historical and future climate parameters in the study area.

Periods	Tmin/°C		Tmax/°C		Annual Rainfall/mm		Annual Rad/(MJ·m^−2^)		Atmospheric CO_2_ Concentration/ppm		CH_4_ Fluxes/(kg C·ha^−1^)	
	RCP4.5	RCP8.5	RCP4.5	RCP8.5	RCP4.5	RCP4.5	RCP4.5	RCP8.5	RCP4.5	RCP8.5	RCP4.5	RCP8.5
1970s	−3.8		8.62		463.65		5091.2		326		125.8	
1990s	−2.6		8.85		544.36		4981.97		353		146.42	
2010s	−2.05		9.14		523.4		4943.33		385		170.57	
2030s	−1.8	−1.67	9.9	9.96	551.84	548.31	4856.25	4835.92	435	449	182.72	190.76
	(+0.65)	(+0.78)	(+0.96)	(+1.02)	(+13.47)	(−9.94)	(−153.69)	(−174.02)	(+83)	(+97)	(+36.76)	(+44.80)
2050s	−1.15	−0.47	10.53	11.14	552.75	573.49	4901.78	4882.47	487	541	218.88	247.16
	(+1.30)	(+1.98)	(+1.59)	(+2.20)	(+14.38)	(+35.19)	(−108.16)	(−127.47)	(+135)	(+189)	(+72.92)	(+101.20)
2070s	−0.55	1.26	11.11	12.8	576.87	603.06	4888.5	4874.42	524	677	238.76	318.65
	(+1.90)	(+3.71)	(+2.17)	(+3.86)	(+38.50)	(+64.69)	(−121.44)	(−135.52)	(+172)	(+325)	(+92.80)	(+172.69)

Note: The values in parentheses indicate the difference between the corresponding values and the mean of 1970s–2010s (i.e., background values). The “+” indicates an increase, and the “−” indicates a decrease.
